# Real-time identification of malignant breast tissue during electrosurgical resection

**DOI:** 10.1038/s41598-026-54277-7

**Published:** 2026-06-16

**Authors:** Selin Guergan, B. Boeer, G. Helms, C. Roehm, V. Bahlinger, I. Gonzalez-Menendez, A. Staebler, K. A. Boehme, A.-S. Haemmerle, S. Nagel, S. Brucker, M. Hahn

**Affiliations:** 1 Department of Women’s Health, Tuebingen University Hospital, Calwerstr. 7, 72076 Tübingen, Germany; 2 Institute of Pathology and Neuropathology, Tuebingen University Hospital, Liebermeisterstr. 8, 72076 Tübingen, Germany; 3 Erbe Elektromedizin GmbH, Waldhoernlestr. 17, 72072 Tübingen, Germany

**Keywords:** Optical emission spectroscopy, Breast cancer, Tumor margin, Machine learning, Support vector machine, Electrosurgery, Cancer, Medical research, Oncology

## Abstract

Achieving complete resection of breast cancer with clear margins remains a significant surgical challenge, particularly for infiltrating subtypes, where re-resection rates of up to 45% have been reported. Consequently, a device capable of providing real-time feedback to surgeons regarding the resected breast tissue holds the potential to significantly improve R0 resection rates. This study represents a further advancement toward intraoperative, real-time classification of breast tissue using optical emission spectroscopy (OES). The objective was to establish the feasibility of OES for distinguishing between normal and pathological breast tissue during electrosurgical incision. Spectra obtained from specimens of 80 patients were analyzed, including 68 patients who underwent breast cancer surgery and 12 patients who underwent risk-reducing or breast reduction surgery. Spectroscopic classification was performed of spectra from tumors with no special type (NST) invasive-lobular carcinoma (ILC) using a machine learning approach based on selected spectral features. The true positive rate reached 91,0% for NST spectra and 78.7% for ILC spectra with true negative rates of 95,2% for NST and 85,4% for ILC. In summary, the current support vector machine (SVM) algorithm demonstrates reliable classification performance for the predominant NST subtype. However, the accuracy of the ILC classification still needs to be improved. Further refinement of the OES-based classification approach is necessary to enhance its reliability across all breast cancer subtypes, particularly the rarer forms, thereby facilitating robust real-time detection during surgery.

## Introduction

Breast cancer is the most commonly diagnosed malignancy in women worldwide, representing approximately one in four cancer cases^1^. While predominantly affecting women, breast cancer can also occur in men, although this accounts for less than 1% of all diagnoses^2^. The global incidence of breast cancer continues to rise, underscoring the need for effective diagnostic and treatment strategies^3,4^.

Surgical excision of the tumor is a cornerstone of early-stage breast cancer treatment. Achieving clear surgical margins is the gold standard recommended by international guidelines, as it significantly impacts patient outcomes^5–9^. However, re-resection due to positive margins is necessary in approximately 16–23% of breast cancer patients^10–12^. This rate is even higher among patients with invasive lobular carcinoma (ILC), ranging from 24% to 45%^12–14^.

Studies underscore the critical importance of achieving complete tumor resection in a single surgical procedure, as positive or close margins have been linked to higher rates of local and distant recurrence, reduced overall survival, and a need for re-excision. Re-excisions not only diminish recurrence-free survival rates but also negatively impact patients’ quality of life and increase healthcare costs^3,15^. Furthermore, re-resections occupy surgical slots that could be used for primary patients, leading to longer waiting times between diagnosis and initial surgery, potentially delaying critical treatment for others.

All electrosurgical resection and coagulation techniques rely on spark generation, with the resulting tissue effects determined by factors such as power output, waveform modulation, peak voltage, duration of exposure, and the type of active electrode, in addition to the intrinsic properties of the tissue^16,17^. During electrosurgery, an electric field is created, leading to the formation of a plasma composed of positively and negatively charged ions, electrons, free radicals, and excited atoms^18^.

The use of electrosurgical tools, e.g. monopolar cutting electrodes or bipolar forceps, is a standard of care in breast surgery. They enable precise tissue dissection while simultaneously achieving haemostasis, thereby reducing intraoperative bleeding, shortening operative time, and enhancing visualization of the surgical field^19^. However, their use is not without limitations. Electrosurgical techniques are associated with the production of surgical smoke and carry a risk of unintended collateral thermal injury to surrounding tissues. Moreover, monopolar electrosurgery presents a potential risk of electromagnetic interference with implanted electronic devices, attributable to the passage of electrical current through the patient. To mitigate occupational exposure to surgical smoke, the incorporation of handpieces equipped with integrated smoke evacuation systems has become an established and widely adopted practice.

Optical emission spectroscopy (OES) is a tool that enables both qualitative and quantitative analysis of the chemical elements and electrolytes released from tissue during electrosurgical procedures. When atoms in the tissue are excited by the energy input from the electrosurgical instrument, they emit characteristic electromagnetic radiation. This radiation, consisting of photons with specific wavelengths, can be detected and analyzed using an optical spectrometer, providing valuable insights into tissue composition^18,20^.

The feasibility of OES for tissue differentiation during monopolar electrosurgery has been successfully demonstrated in malignant and healthy human kidney tissues^18,21^, distinct layers of the human gastric wall^22^, and both healthy and malignant human breast tissues^23^.

In this study, OES was employed to analyze breast tissue from patients with no special type (NST) and ILC during electrosurgical resection. Abnormal breast tissue was defined to include NST, ILC, tumor necrosis, DCIS, LCIS, and tumor-associated stroma, while normal breast tissue encompassed adipose tissue, connective tissue, as well as ductal and glandular structures. The primary objective was to establish a real-time method for differentiating between normal and abnormal breast tissue during dynamic radiofrequency (RF) application as it is performed during electrosurgical tissue incision in the operating room.

## Materials and methods

For the ex vivo study, native human breast tissue samples were obtained from a total of 81 patients treated at the Department of Women’s Health, University Hospital Tübingen between October 2020 and September 2021. Inclusion criteria initially required patients to be over 18 years of age with invasive breast tumors measuring ≥ 20 mm, as determined by preoperative imaging, and no prior endocrine therapy or neoadjuvant chemotherapy. Following a study amendment, the inclusion criteria were expanded to also encompass patients with pT1 tumors and ductal carcinoma in situ, as well as individuals who had received prior treatment (endocrine or chemotherapy). Additionally, benign breast tissue from 12 patients who underwent risk-reducing mastectomies or breast reduction surgeries was analyzed to support the development of the spectral database. One patient in the malignancy group dropped out due to data storage anomaly during the recording process.

To ensure high data quality, the classification analysis in this publication focuses on the two most common subtypes, NST and ILC.

All patients provided written informed consent for the use of their tissue samples. All research was performed in accordance with relevant guidelines as well as the Declaration of Helsinki.

The study has been approved by the Ethics Committee of the Tuebingen University Hospital, project number 254/2017BO2, and registered at the German Clinical Trials Register (DRKS00012767, https://drks.de/search/en/trial/DRKS00012767/details; registration date 02/07/2018).

The primary aim of this study was to establish OES tissue discrimination (normal vs. abnormal) during electrosurgical resection of human breast tissue, which is the planned clinical use of the final OES-RF-device. Furthermore, the classification algorithm was refined with additional data, and the accuracy of classification was evaluated in confusion matrices for the group of NST and ILC patients as well as the whole cohort.

**Fig. 1:**
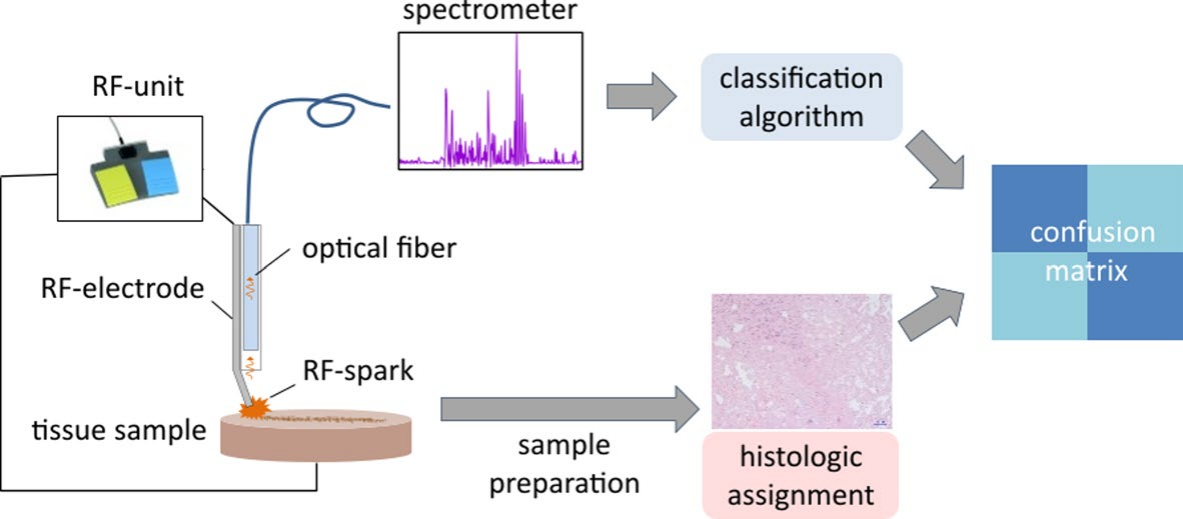
Setup of OES measurements with ex vivo breast tissue samples: A radiofrequency (RF) current was applied to the tissue via an active RF-spatula electrode, generating short electric arcs (sparks) that excite atoms and molecules within the tissue. The emitted light (optical emission) was collected through an optical fiber and analyzed using an optical emission spectrometer. Diagnostic performance was evaluated by comparing spectral classifications to histological reference diagnoses using a confusion matrix, quantifying true positive, true negative, false positive, and false negative rates.

Figure 1 summarizes the setup of the study.

### Measurement of optical emission spectra

The experimental setting included a prototype instrument containing an active spatula electrode for RF-incision and an optical fiber to collect and transfer the emitted light to a spectrometer. For RF-incision, an electrosurgical unit (VIO3, Erbe Elektromedizin GmbH, Germany) was used. Optical emission spectra were recorded with a Maya2000Pro spectrometer (Ocean Optics Germany GmbH, Germany) set to an integration time of 10ms (20 spectra of 10ms each were summed up to one 200ms spectrum). During each incision several sum spectra were recorded. Potential contamination of the optical fiber by tissue aerosol was monitored by determination of transmission quality using a deuterium/halogen lamp (Ocean Optics Germany GmbH, Germany) and a Maya 2000Pro spectrometer after each incision.

### Histopathological evaluation

**Fig. 2:**
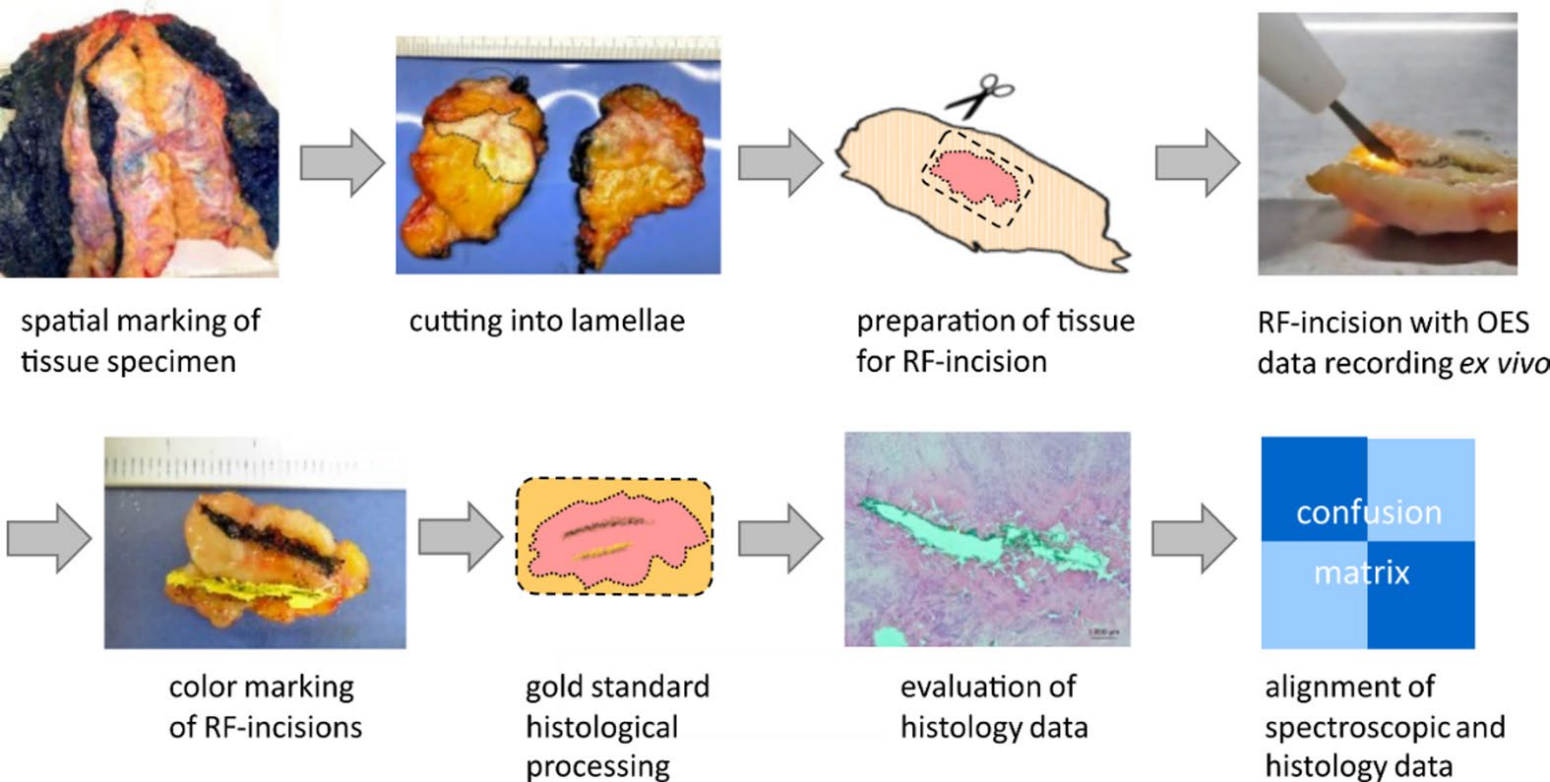
Workflow of ex vivo breast tumor specimen preparation, RF-OES device incision, histological processing and data alignment. The black marking on the cut lamella indicates an area of abnormal tissue. The tumor tissue shown in the tissue samples derives from a NST breast cancer patient.

The fresh resected breast cancer specimens underwent precise topographic orientation and marking with permanent tissue color and were cut into lamellae. Parts of selected lamellae dispensable for diagnosis including normal and/or abnormal tissue underwent RF-incisions with OES data recording (Fig. 2). To start spectral recording, the spark detection sensor of the VIO3 was used as trigger. After OES examination of the tissue samples, the precise locations of RF-incision and spectra generation were permanently inked and photo documented. Subsequently, the tissue samples were fixed in 4.5% neutral buffered formalin, embedded in paraffin and serial sectioning spaced approximately 100–120 μm apart was performed for the whole RF-incision cutting area. All sections were stained with hematoxylin and eosin (HE) and evaluated by a pathologist and a second trained observer in a blinded fashion as standard of practice. The percental portion of normal and abnormal tissue was determined for all made incisions. Invasive tumor (NST, ILC), tumor necrosis, DCIS, LCIS and tumor stroma were considered abnormal tissue, whereas normal breast parenchyma contained adipose tissue, glandular tissue and connective tissue. For final evaluation, histologically normal or abnormal tissue classification was evaluated against the classification based on corresponding recorded OES spectra.

### Signal preprocessing and spectroscopic features for tissue differentiation

The recorded optical emission spectra were baseline corrected using the asymmetric least square algorithm or a linear fit. Subsequently, the spectra were evaluated by a quality criterion set based on the strengths of carbon emission lines in relation to the background noise. Only spectra passing the quality criterion were considered for further analysis. To compare the spectra with each other, they were normalized to their individual total intensity. After preprocessing, the spectra were used for classification.

### Classification of spectroscopic features and machine learning

The classification of spectra from normal and abnormal breast tissue was done by a Support Vector Classifier (SVC) trained with features unique to normal and abnormal tissue extracted from optical emission spectra with a sufficient signal-to-noise ratio and a minimum occurrence rate of more than 5%^23^. Prediction correctness was determined, and results were annotated in confusion matrices.

Data analysis was performed using custom software written in Python 3.6.8 with NumPy 1.19.5, Pandas 1.1.5, Scikit-learn 0.24.2, and SciPy 1.5.4 as well as a set of non-commercial libraries^24^.

## Results

### Patients and tissue samples

The analysis included 80 patients with a total of 602 RF-OES incisions made on tumor specimens. The mean age of the cohort was 58 years (± 15), with only one male patient.

68 patients underwent oncological breast surgery, whereas 12 patients underwent risk-reducing or breast reduction procedures.

The most common pathological tumor stage was pT2, accounting for 50% of patients and 51,2% of incisions. Lower stages (pTis and pT1) were less frequent, while higher stages (pT3 and pT4) were rare. Most tumors were graded as G2 (61,7% of patients and 62,1% of incisions), indicating moderate differentiation. G3 tumors, which are poorly differentiated, accounted for 35,7% of patients and 36,1% of incisions. HR (hormone receptor)-positive tumors made up 91,2% of the malignant tissue. Her2/neu status was predominantly classified as low (64.7% of patients and 68.7% of incisions), with fewer cases being Her2/neu negative or positive. Regarding histological subtype, invasive carcinoma of no special type (NST), either alone or in combination with ductal carcinoma in situ (DCIS), constituted the majority of cases (47%). Additional subtypes included lobular carcinoma in situ (LCIS), in both classic and pleomorphic forms, which collectively accounted for 23% of patients.

Surgical interventions included breast-conserving surgery in 35% of cases, primary mastectomy in 28%, and oncologic nipple- or skin-sparing mastectomy (NSM/SSM) in 21%. In addition, risk reducing procedures and reduction mammoplasties were included for OES data acquisition in benign tissue.

The majority of patients (78%) did not receive preoperative therapy. Neoadjuvant chemotherapy was administered in 15% of cases, while 7% of patients underwent primary endocrine therapy.

An overview of patient and tissue characteristics can be seen in Tables 1 and 2.

**Table 1:** Tumor and patient characteristics.

Tumor characteristics	*N* (100%) Total *N* = 80 Patients	*N* (100%) Total *N* = 602 incisions
Age mean (± SD)	58 (± 15) years	
Sex		
Female	79 (99%)	599 (99%)
Male	1 (1%)	3 (1%)
Histological subtype	80 (100%)	602 (100%)
Benign	12 (15%)	140 (23%)
DCIS	5 (6%)	36 (6%)
NST	9 (12%)	43 (7%)
NST+DCIS	28 (35%)	196 (33%)
NST + Other	1 (1%)	5 (1%)
ILC	6 (7%)	38 (6%)
ILC+ LCIS + DCIS	1 (1%)	14 (2%)
ILC + LCIS	16 (20%)	113 (19%)
Others	2 (3%)	17 (3%)
LCIS, classic type	15 / 17	
LCIS, pleomorphic type	2 / 17	
Operation types	80 (100%)	602 (100%)
Breast conserving surgery	28 (35%)	152 (25%)
Primary mastectomy	23 (29%)	188 (32%)
Oncologic NSM/SSM	17 (21%)	122 (20%)
Risk reducing NSM	4 (5%)	45 (8%)
Risk reducing mastectomy	1 (1%)	15 (2%)
Reduction surgery	7 (9%)	80 (13%)
Preoperative therapy	80 (100%)	602 (100%)
None	62 (78%)	501 (83%)
Primary endocrine therapy	6 (7%)	44 (8%)
Neoadjuvant chemotherapy	12 (15%)	57 (9%)

SD; standard deviation; DCIS: ductal Carcinoma in situ; NST: no special type; ILC: invasive lobular carcinoma; LCIS: lobular Carcinoma in situ; NSM: nipple sparing mastectomy; SSM: skin sparing mastectomy.

**Table 2:** Characteristics of examined malignant tissue.

Tumor characteristics	Number of patients *n* = 68 (100%)	Number of incisions *n* = 462 (100%)
pT status		
pTis	5 (7,4%)	36 (7,8%)
pT1	7 (10,3%)	52 (11,3%)
pT2	34 (50,0%)	237 (51,3%)
pT3	8 (11,8%)	69 (14,9%)
pT4	2 (2,9%)	11 (2,4%)
ypTis	3 (4,4%)	15 (3,2%)
ypT0	3 (4,4%)	12 (2,5%)
ypT1	3 (4,4%)	16 (3,5%)
ypT2	2 (2,9%)	10 (2,2%)
ypT3	1 (1,5%)	4 (0,9%)
Grading	68 (100%)	462 (100%)
G1	1 (1,5%)	8 (1,7%)
G2	42 (61,7%)	287 (62,1%)
G3	25 (36,8%)	167 (36%)
HR status	68 (100%)	462 (100%)
HR positive	62 (91,2%)	431 (93,3%)
HR negative	6 (8,8%)	31 (6,7%)
Her2/neu status	63 (79%)	426 (71%)
Her2/neu negative	14 (18%)	91 (15%)
Her2/neu low	43 (54%)	298 (50%)
Her2/neu positive	6 (%)	37 (6%)

To evaluate the performance of OES on untreated tissue, dynamic incisions were performed on samples from 62 patients who had not received any prior therapy.

**Fig. 3:**
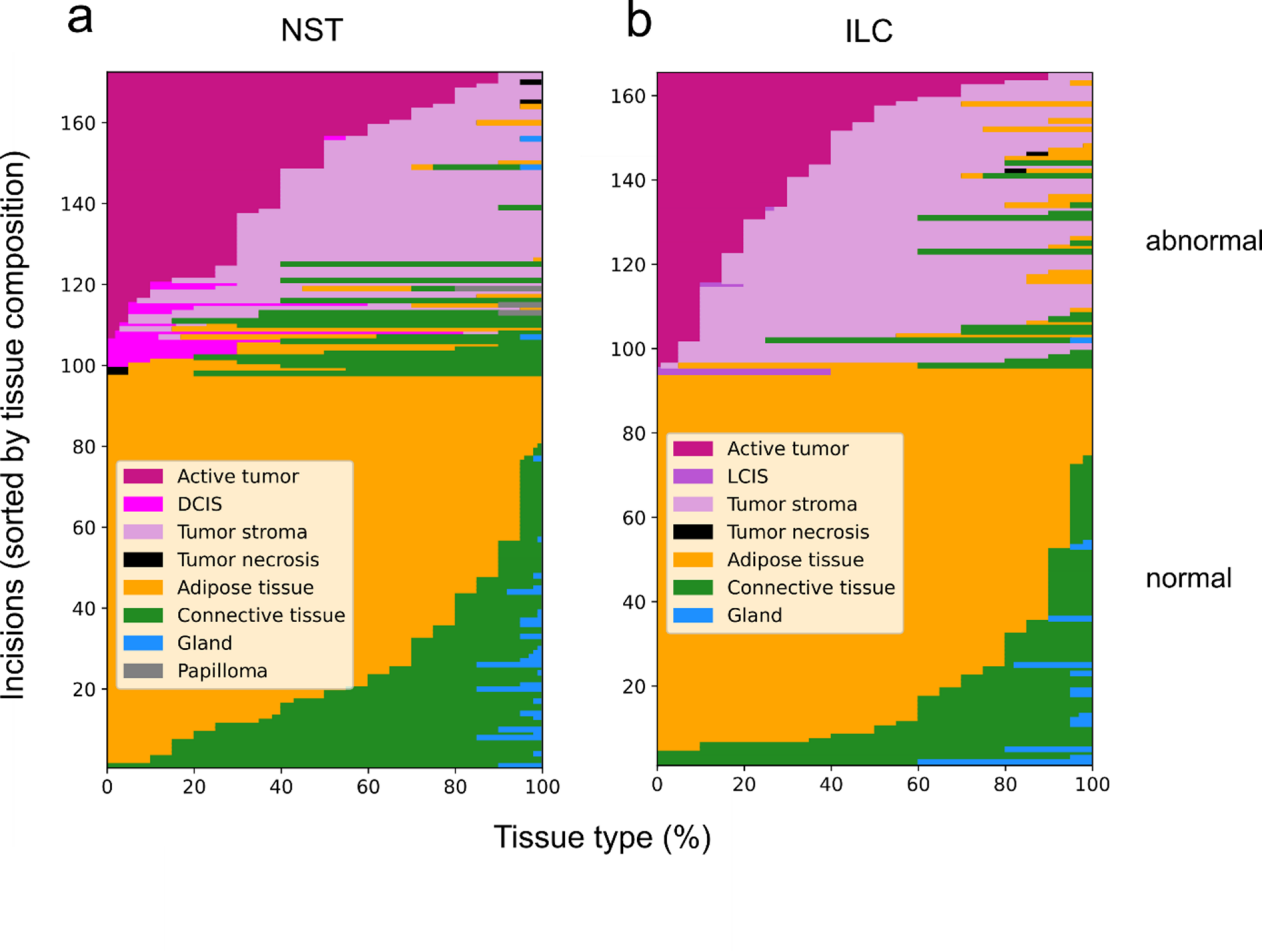
Tissue type distribution across incisions labelled according to histopathologic analysis. For the (a) 26 NST-Group and (b) 23 ILC-Group patients without prior treatment included in the study, all tissue types present in individual incisions have been sorted by tissue composition. All incisions presenting active tumors (NST or ILC depending on group), DCIS, LCIS, tumor stroma or tumor necrosis are rated as abnormal, whereas incisions including only adipose tissue, connective tissue and glands are summarized as normal tissue. Other tissue detected in incisions of the NST group consists of papilloma tissue derived from one patient.

In the NST group, a total of 182 incisions of tissue were analyzed, including 81 incisions made on abnormal tissue. In the abnormal tissue group, 71/81 (87,7%) incisions contained active NST tumor, 9/81 (11,1%) incisions exhibited combined NST and DCIS, and 7/81 (8,6%) contained only DCIS. Additionally, 4/81 (4,9%) incisions included tumor necrosis, with 2/81 (2,5%) incisions containing only necrosis. Tumor stroma was present in 71/81 (87,7%) incisions in abnormal tissue. In the normal tissue group, incisions predominantly contained fat tissue, which was absent in only 2/101 (2,0%). Connective tissue was present in 86/101 (85,1%) incisions, and glandular tissue in 27/101 (26,7%). One patient in the NST cohort had abundant intraductal papilloma tissue (Fig. 3a).

In the ILC group, 164 incisions were analyzed, with 71 containing abnormal tissue (Fig. 3b; Table 2). Active ILC tumor was present in 70/71 (98,6%) of these incisions. ILC combined with LCIS was observed in 2/71 (2,8%) incisions, and LCIS alone in another 2/71 (2,8%). Tumor stroma was found in 70/71 (98,6%) incisions in abnormal tissue. A total of 93 incisions containing normal tissue were analyzed, with adipose tissue found in 89/93 (95,7%) incisions, connective tissue in 71/93 (76,3%), and glandular tissue in 13/93 (14,0%). Based on the evaluation of tissue type distribution across incisions made in both groups, NST and ILC were similar.

### Selected features for machine learning

**Fig. 4:**
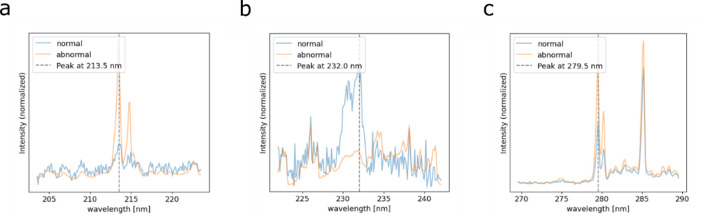
Overview of the three selected features for tissue classification. The spectral peaks at (a) 213.5 nm (phosphor/zink), (b) 232.0 nm (carbon) and (c) 279.5 nm (magnesium) were used for tissue classification. The figure shows the mean spectra of normal and abnormal tissue derived from the 26 patients with NST tumors. Spectra were normalized for the overlay due to different spectral intensities in normal and abnormal tissue.

The feasibility study for OES breast tissue analysis employed a high-resolution Echelle spectrometer and identified a total of 19 discriminative spectroscopic features across the entire spectral range^23^. In contrast, the present study utilized a more cost-effective, lower-resolution optical emission spectrometer (OceanOptics Maya2000 Pro) with a narrower optical window (200–424 nm), selected for its suitability in future clinical applications. Therefore, some of the previously identified features are no more available for analysis. The three features shown in Fig. 4 were selected for tissue classification.

### Histological differences of NST and ILC tumors

**Fig. 5:**
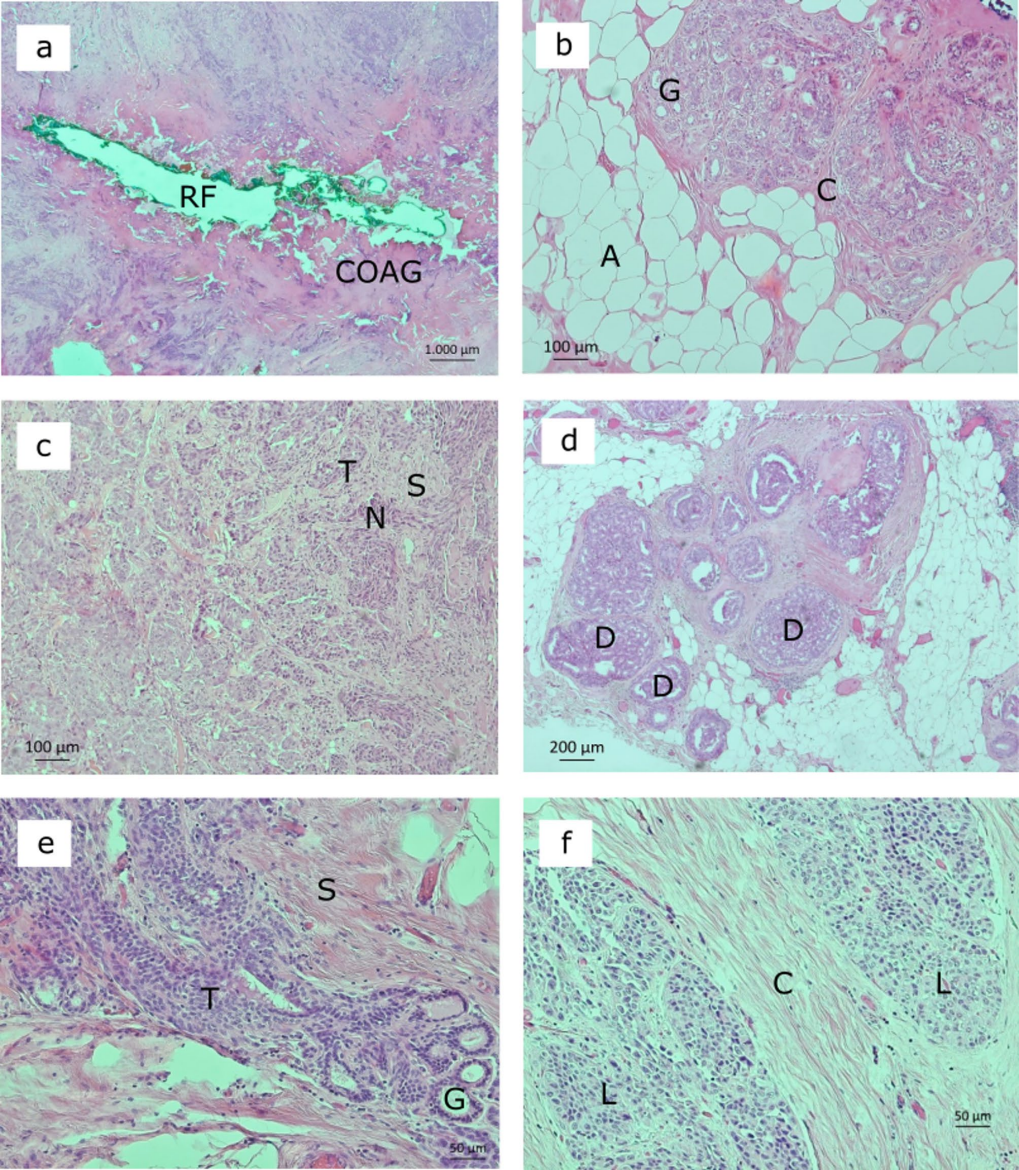
Typical histology of (a) RF-incision in NST tumor tissue, (b) normal breast tissue, (c) NST tumor, (d) DCIS, (e) ILC tumor, (f) LCIS. Abbreviations: Adipose tissue (A), coagulated tissue (COAG), connective tissue (C), duct with DCIS (D), gland (G), LCIS (L), necrosis (N), RF-incision with green ink (RF), tumor stroma (S), tumor (T).

Figure 5 presents HE-stained tissue sections, offering an overview of the entire RF-incisions and specific tissue types. The RF-incision surface, depicted in Fig. 5a, is marked with green tissue ink, highlighting the vacuolized and coagulated tissue surrounding the incision. This coagulation results from the heat generated during monopolar RF resection. Pathologists classified the adjacent normal and abnormal tissue content surrounding the incision.

For comparative purposes, the typical histological features of normal (Fig. 5b) and abnormal (Fig. 5c-f) breast tissue (NST, DCIS, ILC and LCIS, respectively) are illustrated. NST (Fig. 5c) is characterized by pleomorphic tumor cells arranged in nest-like structures accompanied by necrotic areas and reactive tumor stroma. In contrast, ILC (Fig. 5e, compare scale bar) exhibits filamentous growth of small, irregularly shaped tumor cells into a fibrous stroma. DCIS cells (Fig. 5d) fill the mammary ducts but show no invasion into adjacent tissue. LCIS (Fig. 5f) forms in the terminal ductal lobular unit (TDLU), filling acini, which are glandular tissues involved in milk production. By definition, LCIS is diagnosed when more than 50% of the acinar tissue is filled and expanded by tumor cells^25^.

### Classification accuracy based on tumor type

**Fig. 6:**
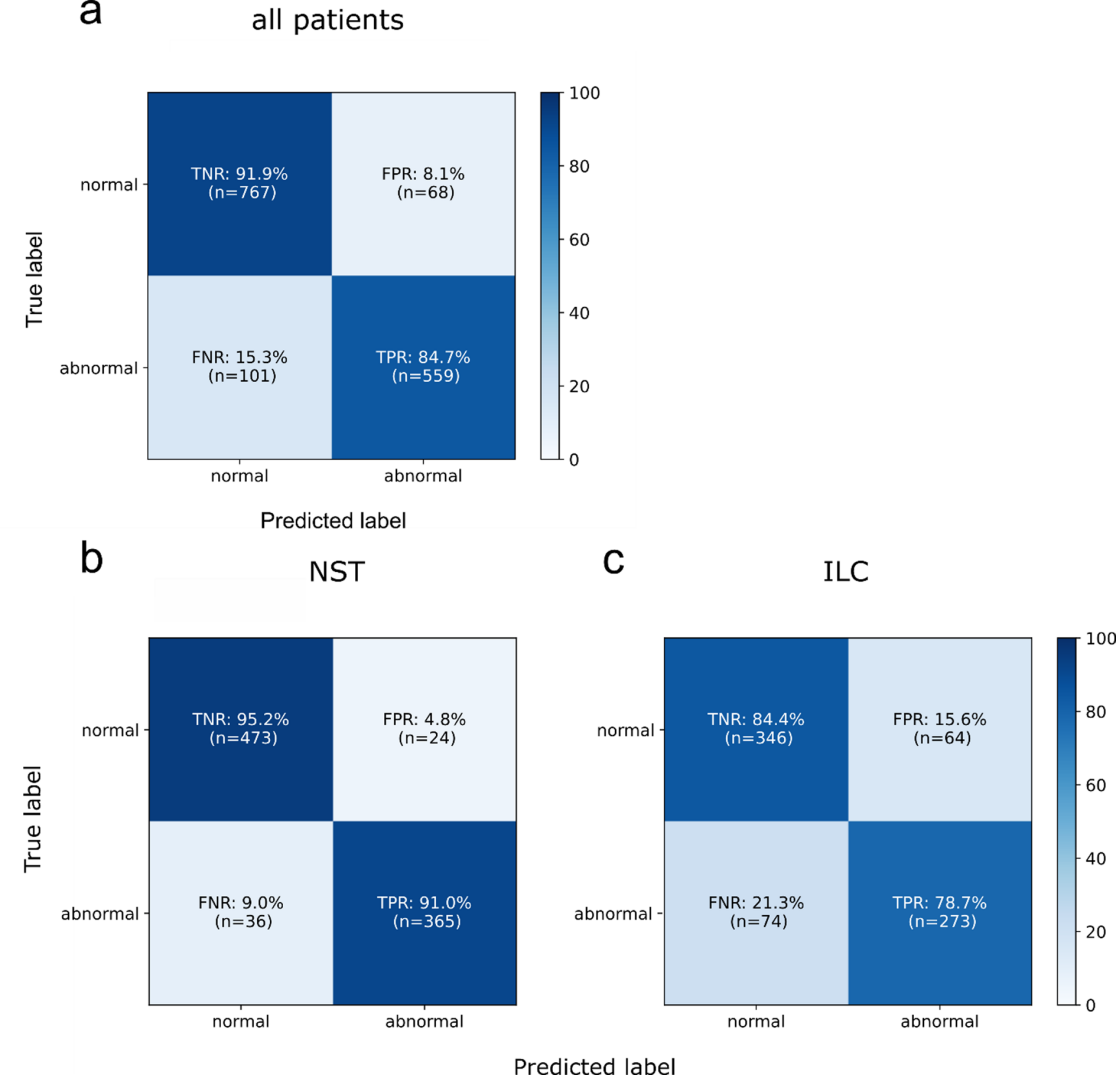
Confusion matrices showing true positive (TPR), true negative (TNR), false positive (FPR) and false negative (FNR) classification of spectra in (a) all patients, (b) NST (*n* = 26) and (c) ILC (*n* = 23) patients. The number (n) of spectra allocated to the individual groups is shown in brackets. Correct prediction is depicted by a color range.

The confusion matrices show the true positive, true negative, false positive and false negative classification of spectra in NST and ILC patient subgroups (Fig. 6).

In the entire patient cohort (Fig. 6a), the SVC achieved a true negative rate of 91,9% and a true positive rate of 84,7%. The false positive and false negative rates were 8,1% and 15,3%, respectively. These results indicate high specificity and good sensitivity in distinguishing all normal from abnormal tissue.

For the NST cohort (Fig. 6b), 473/497 (95,2%) spectra were correctly classified as normal tissue, while 365/401 (91,0%) spectra were accurately classified as abnormal tissue. Additionally, 36/401 (9,0%) spectra were classified as false negatives, and 24/497 (4,8%) were classified as false positives. Notably, all 21 spectra obtained from the male NST patient were correctly classified with 100% accuracy.

For the ILC cohort (Fig. 6c), 346/410 (84,4%) spectra were correctly classified as normal tissue, and 273/347 (78,7%) were correctly classified as abnormal tissue. The false negative rate for ILC was 74/347 (21,3%), and the false positive rate was 64/410 (15,6%).

## Discussion

Numerous studies and clinical guidelines emphasize the critical importance of achieving R0-resections. Moreover, the incidence of breast cancer cases is increasing^1–4,6,7,9,10^. In this context, the optimization of real-time differentiation between normal and abnormal breast tissue during surgery is paramount for enhancing patient care and improving surgical outcomes.

Margin assessment is crucial not only for improving patient survival and reducing recurrence rates but also for benefiting other patients by shortening waiting times for surgery and easing the burden on the healthcare system by lowering costs^3,15^. Over the years, various techniques have been introduced to reduce R1 rates^1,6,9,26^.

A range of techniques has been developed for intraoperative margin assessment, including Margin Probe, ClearCoast, intraoperative ultrasound (IOUS), micro-CT, and intraoperative MRI, with continuous advancements in the field^26–31^. Given its considerable clinical significance, research and development in this area remain ongoing^14^. Among these modalities, IOUS has been successfully integrated into clinical practice, with meta-analyses demonstrating its efficacy in reducing R1 rates^28^. Notably, these techniques primarily assess surgical margins at a macroscopic level through diagnostic imaging. In contrast, OES enables tissue evaluation at a microscopic, cellular level, offering a different dimension of intraoperative assessment.

OES operates exclusively during HF-mediated tissue transection. The system is designed as an intraoperative adjunct in breast cancer surgery, delivering real-time feedback directly at the dissection interface without disrupting the procedural workflow. It is not intended to guide preoperative or intraoperative planning of resection extent, to reduce surgical margin width, or to replace established margin assessment methods, including the histopathological evaluation of excised tissue specimen.

In addition, also intraoperative cytologic examination of tumor margins in resected specimens is established. Frozen section pathology is primarily utilized for assessing sentinel lymph nodes. However, its effectiveness in detecting and confirming marginal involvement is limited, with a notable risk of false-negative and false-positive results. These inaccuracies can lead to unnecessary resection of healthy tissue or incomplete tumor removal, compromising surgical outcomes^26,32^.

Similarly, cytology offers cellular-level margin assessment and, along with frozen section analysis, has demonstrated high diagnostic accuracy in meta-analyses. However, both methods must be evaluated in the context of their resource-intensive nature, including the need for an on-site pathologist, which is not always feasible^26^. Additionally, frozen section analysis prolongs anesthesia and surgical duration, increasing clinical, economic, and social costs while contributing to longer waiting times for other patients^26,33^. NST tumors, which account for 75–80% of breast cancer cases worldwide^11,12^, were classified using OES with high specificity and sensitivity, demonstrating its effectiveness for this subtype.

ILC, which represents 10–15% of cases^11,12^ with its filamentous growth pattern and non-cohesive infiltration into surrounding tissue caused by E-cadherin loss, provides a challenge with higher re-resection rates (24–45%) compared to NST (16–23%) and other breast cancers^6,34,35^. This underscores the critical need for improved intraoperative margin assessment techniques for ILC to reduce reoperation rates and enhance patient outcomes.

In the ILC group, the OES classification algorithm identified 21.3% of spectra as false negatives, which is higher than for NST but still lower than the reported re-resection rates. In clinical practice, this could result in R1-resections if not detected by the OES device. Also, the false positive rate for ILC (15,6%) was significantly higher than for NST (4,8%), suggesting that the current SVC algorithm may be less effective for ILC than for NST.

Due to the complex growth pattern of ILC, its margins are more difficult to detect compared to NST. Moreover, while OES classifies vaporized tissue during RF-resection, histology evaluates the residual post-incision tissue, leading to discrepancies in ground truth identification, particularly when tissue affiliation shifts on a small scale.

It is important to note that the false negative and false positive rates were calculated per spectrum, with each spectrum recorded over 200 ms. If tumor tissue is abundant, subsequent spectra would likely provide more accurate detection as the algorithm receives stronger signals. Additionally, the human response time to optical and acoustic feedback is approximately 200–300 ms^16,17^. Given that ILC typically has less distinct margins, the SVC algorithm may struggle to classify spectra from ambiguous margin regions. This is because the actual algorithm classifies data points based on a binary hyperplane, supporting no unclear classifications. Therefore, the use of an artificial neural network (ANN), capable of analyzing complex correlations, may yield more accurate results, particularly for ILC, as larger datasets become available for training^11^.

Since OES detection relies on the excitation of atoms and molecules during RF-resection, it analyzes the vaporized tissue instead of the surrounding tissue that is examined histologically. This becomes particularly important for ILC, which has poorly defined margins due to non-cohesive tumor cell growth, potentially causing discrepancies between OES and histologic classifications. Furthermore, for tissue types that cannot be clearly classified by SVM, an ANN-based algorithm could offer improved results. ANNs may also provide real-time intraoperative feedback by alerting surgeons when transitional spectra of tissue with unsure features are detected.

While histological classification remains the gold standard for tissue evaluation, OES has the potential to provide intraoperative real-time feedback, optimizing intraoperative decision-making for improved patient outcomes.

## Conclusion

This study represents a significant advancement in real-time differentiation of normal and abnormal breast tissue during dynamic electrosurgical incision by integrating OES-based tissue classification.

Our findings demonstrate that OES can effectively distinguish between benign and malignant breast tissue by analyzing the sparks generated by the electrical scalpel during tissue incision. However, further research is necessary to refine the technique for optimal discrimination across all breast tissue types. The next step involves the clinical translation conducting a first-in-human trial to evaluate the feasibility in a surgical setting.

## Data Availability

The datasets generated and analysed during this study are available from the corresponding author upon reasonable request.
